# Polymicrobial Wound Infection Caused by Lelliottia amnigena, Staphylococcus aureus, and Corynebacterium Following a Lawnmower Accident

**DOI:** 10.7759/cureus.88655

**Published:** 2025-07-24

**Authors:** Richard Giovane, Luis Pernia, Wesley Faught, Pia Cumagen, James M Comer

**Affiliations:** 1 Family Medicine, The University of Alabama, Tuscaloosa, USA; 2 Plastic Surgery, Druid City Hospital (DCH) Regional Medical Center, Tuscaloosa, USA; 3 Family Medicine, Druid City Hospital (DCH) Regional Medical Center, Tuscaloosa, USA; 4 Infectious Disease, Druid City Hospital (DCH) Regional Medical Center, Tuscaloosa, USA; 5 Infectious Disease, University of Mississippi Medical Center, Jackson, USA

**Keywords:** cellulitis, corynebacterium, lelliottia amnigena, methicillin-resistant staphylococcus aureus, traumatic injury

## Abstract

*Lelliottia amnigena*, a Gram-negative, facultatively anaerobic bacterium commonly isolated from aqueous and soil environments, is typically an opportunistic pathogen, particularly in immunocompromised hosts. Despite its low prevalence as a human pathogen, documented cases include urinary tract infections, acute cholecystitis, and sepsis. In contrast, methicillin-resistant *Staphylococcus aureus* (MRSA), a coagulase and catalase-positive Gram-positive organism, is frequently associated with a spectrum of infections, ranging from skin infections to severe conditions such as pneumonia and endocarditis. Furthermore, pathogenic species of *Corynebacterium*, including *Corynebacterium diphtheriae*, are recognized for their role in upper respiratory infections and other serious conditions. We report a novel case of cellulitis due to the coinfection of *L. amnigena*, MRSA, and *Corynebacterium* in a patient who suffered a trauma to three toes from a lawnmower incident. Notably, the patient possessed no prior medical history or discernible immunocompromising factors, underscoring the unusual nature of this presentation. This case emphasizes the importance of obtaining intraoperative cultures and employing culture-directed antimicrobial therapy in managing complex, environmentally contaminated wounds.

## Introduction

*Lelliottia amnigena* is a Gram-negative, facultatively anaerobic, rod-shaped bacterium that is commonly found in water and soil [1]. While historically considered a pathogen rarely isolated in humans, *L. amnigena* is increasingly recognized as a potential cause of infections, particularly in immunocompromised patients, with the literature showing sparse case reports of *L. amnigena* causing urinary tract infections, acute cholecystitis, and sepsis [2,3].

*Staphylococcus aureus*, more specifically methicillin-resistant *Staphylococcus aureus* (MRSA), is a Gram-positive, coagulase and catalase-positive pathogen, which is methicillin-resistant, commonly found on the skin and in the nasal passage [4]. MRSA is a common source of infection and causes a wide range of illnesses, including pneumonia, bacteremia, cellulitis, and endocarditis [5]. Moreover, MRSA is commonly found in polymicrobial infections and can pose a challenge for clinicians to treat effectively [4,5].

*Corynebacterium* species are gram-positive, catalase-positive, non-spore-forming rods that are subdivided into pathogenic and non-pathogenic species [6]. Pathogenic species of *Corynebacterium* are known to cause upper respiratory tract infections, most notably diphtheria from *Corynebacterium diphtheriae*, urinary tract infections, bacteremia, osteomyelitis, and endocarditis [7]. Like MRSA, *Corynebacterium* is also common in polymicrobial infections.

We present a novel case of an individual who presented to the hospital after a lawnmower blade lacerated his right foot. This caused a comminuted fracture of the great toe with non-displaced fractures of the second and third toes. This leads to a complete amputation of his second and third digits and causes local cellulitis from *L. amnigena*, MRSA, and *Corynebacterium*.

## Case presentation

We report a case of a 62-year-old male patient with a past medical history of lumbar radiculopathy and carpal tunnel syndrome, who presented to the emergency department following a traumatic lawnmower accident. The patient reported losing control of the mower while pushing it up a steep hill, resulting in a laceration to his right foot. He presented to the emergency room immediately after the incident. He denied pain at the time of evaluation, and his last meal was two hours prior to the incident occurring. The patient reported smoking a pack of cigarettes daily but denied alcohol or illicit drug use.

**Figure 1:**
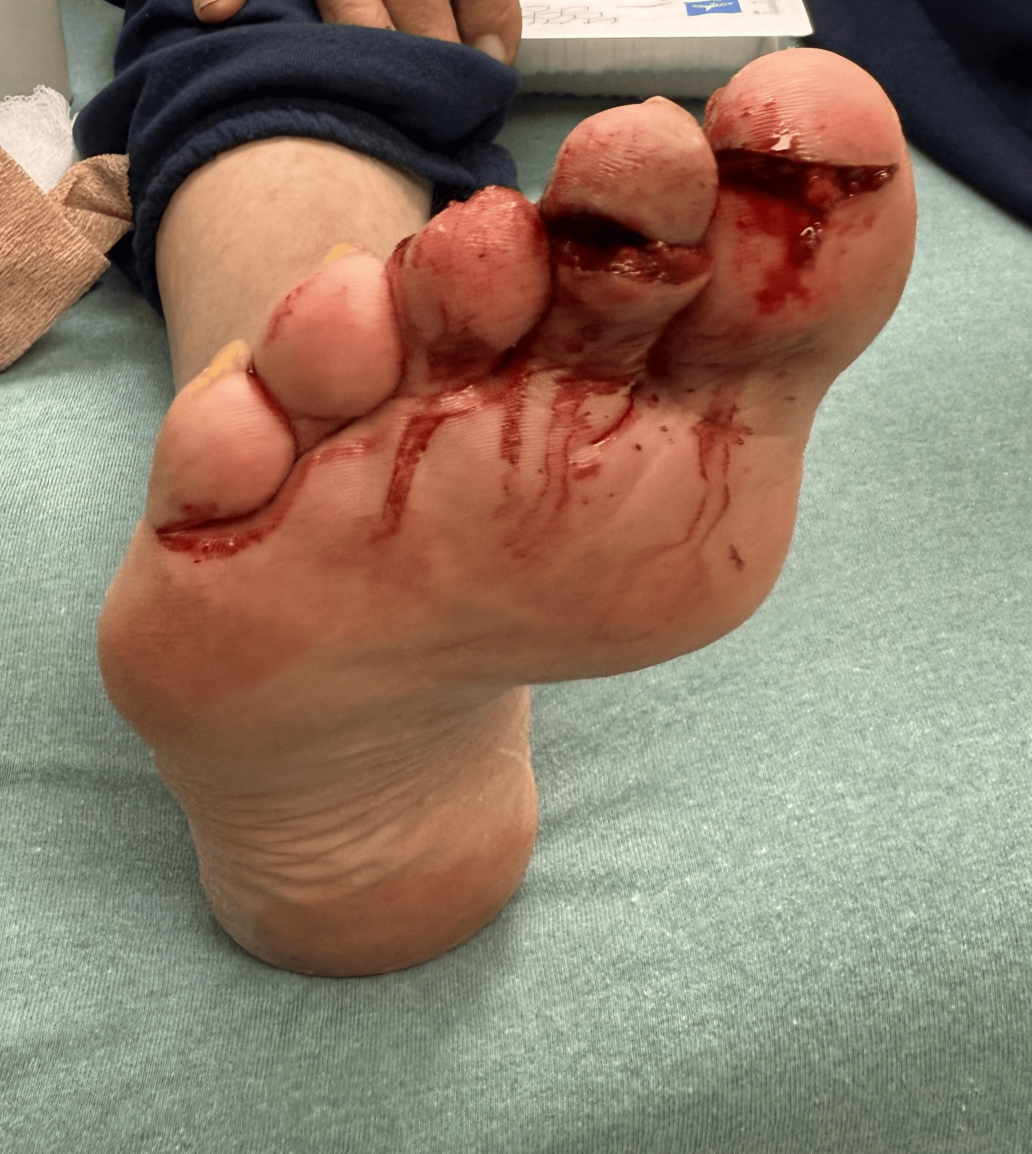
Laceration of the first and second toes

On initial assessment, his vital signs were a temperature of 98°F, heart rate of 88 beats per minute, respiratory rate of 18 breaths per minute, blood pressure of 138/84mmHg, and an O2 saturation of 97% on room air. Upon review of systems, the patient had no pertinent positives. On physical examination, the patient was well-appearing and hemodynamically stable. Deep lacerations were noted with surrounding erythema and mild edema on the plantar region of the first and second toes, extending laterally to the sides of the toe (Figure 1).

**Figure 2:**
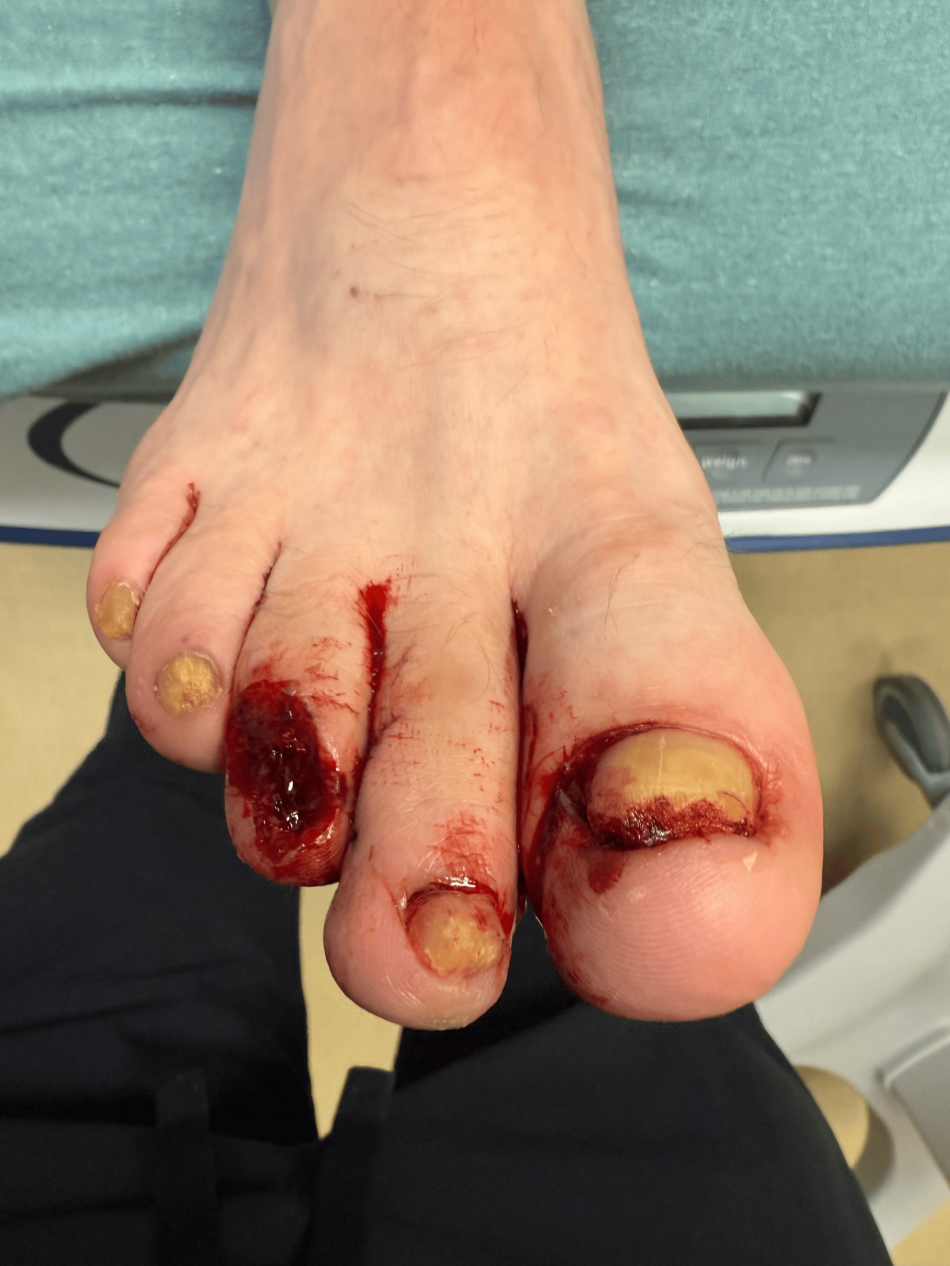
Dorsal view of the laceration on the third toe

An extensive laceration was noted on the nail bed of the third toe (Figure 2).

The right foot was then wrapped with dorsalis pedal pulses 2+ and neurovascularly intact. A complete blood count and complete metabolic panel were ordered, which showed a white blood cell (WBC) count of 10.10 × 10^3^/uL, hemoglobin of 14.1 g/dL, hematocrit of 41.7%, and a platelet count of 264 × 10^3^/uL, and sodium of 148 mEq/L, potassium of 4.2 mEq/L, and glucose of 109 mg/dL.

**Figure 3:**
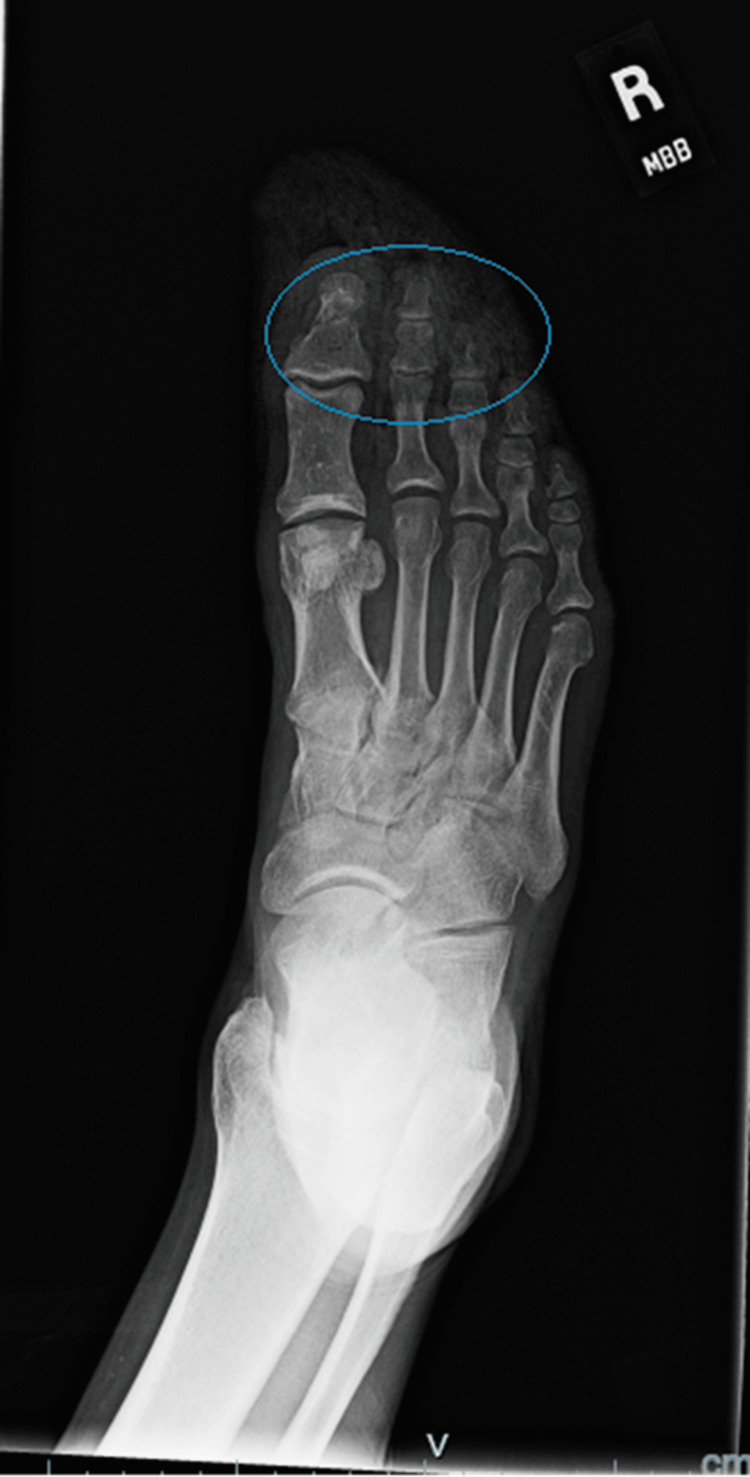
X-ray of the dorsum of the patient's right foot outlining the fractures of all three toes

**Figure 4:**
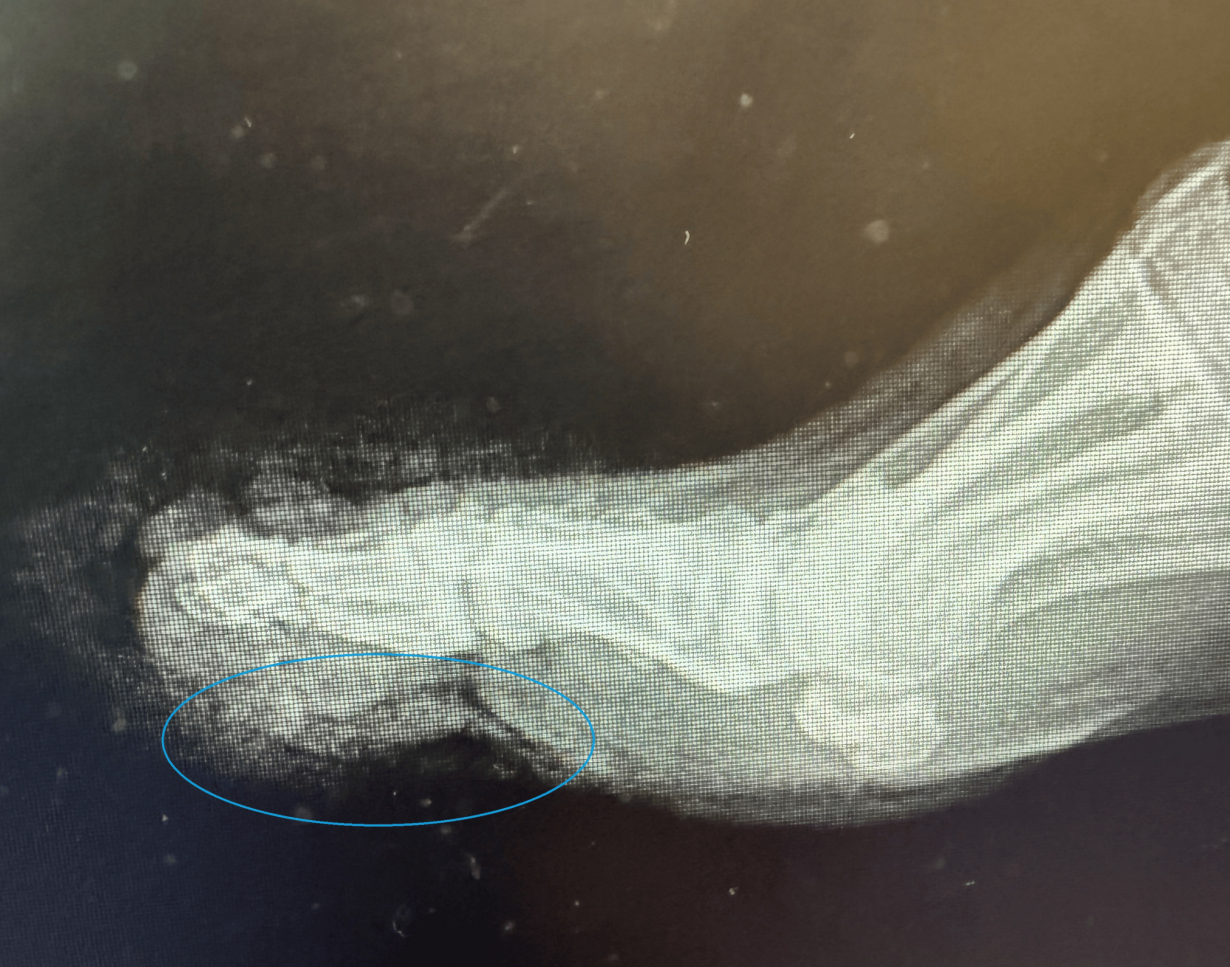
Lateral X-ray outlining extensive laceration to the patient's toes

An X-ray of the patient's foot was taken (Figures 3, 4), which showed a comminuted fracture of the great toe with the metatarsal appearing present. A non-displaced fracture of the tuft of the second toe was also noted, with a fracture of the second toe's middle and distal phalanges. Lastly, a fracture of the third toe distal phalanx was noted.

**Figure 5:**
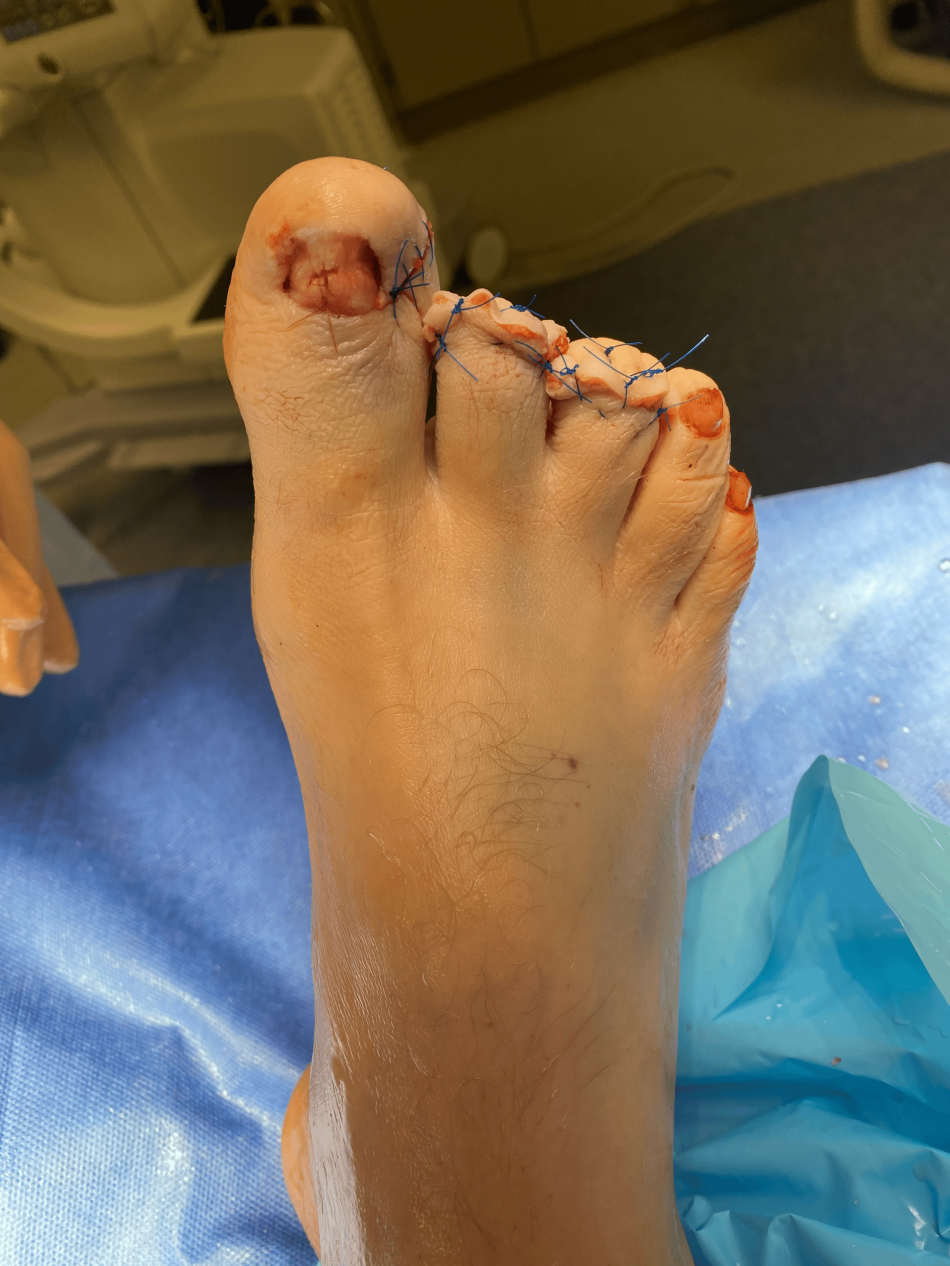
Intraoperative amputation of the second and third toes

**Figure 6:**
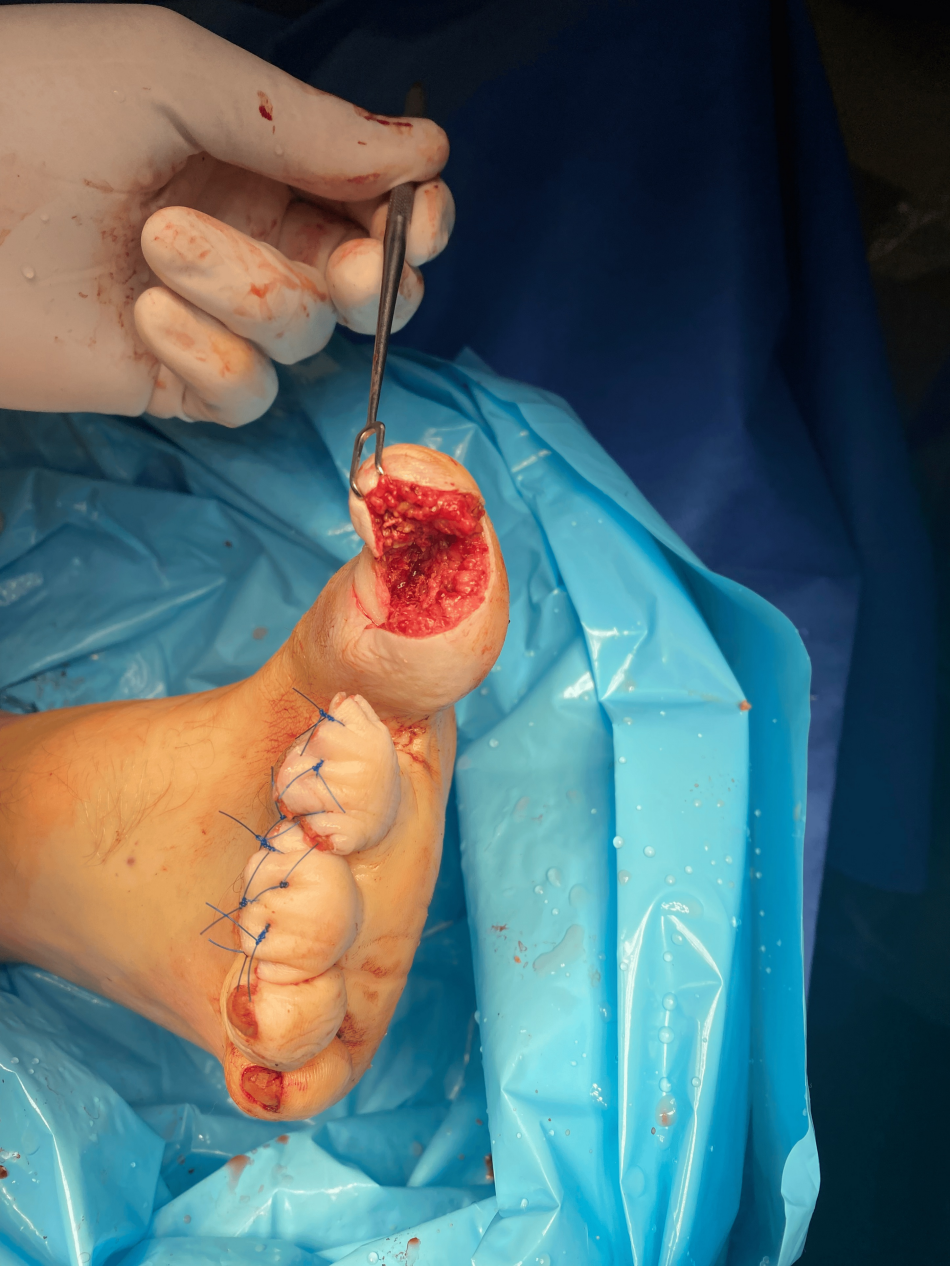
Intraoperative image showing the extent of the laceration of the first toe

The patient was started on IV cefazolin (Ancef) and received tetanus toxoid and tetanus immunoglobulin due to the patient not being up to date on his vaccines. He was evaluated by plastic surgery, who recommended that the patient be taken to the OR for urgent surgical management. The patient underwent successful debridement of the open fractures and an open reduction internal fixation (ORIF) of the first toe, followed by a nail bed repair and partial amputation of the second and third toes (Figures 5, 6).

Cultures of the wound were taken intraoperatively. Postoperatively, IV cefazolin was discontinued, and the patient was placed on IV piperacillin-tazobactam (Zosyn) and vancomycin for broad coverage due to the nature of the injury and extent of damage to the tissue. The patient was monitored postoperatively without any acute complications. Wound cultures grew back *Lelliottia amnigena*,* Corynebacterium* (non-speciated), and* *MRSA. Infectious disease was consulted, who recommended that the patient complete a six-week course of linezolid 600 mg twice daily and amoxicillin/clavulanic acid 875/125 mg twice daily. The patient was monitored for 48 hours total in the hospital, was able to ambulate without issue with physical therapy, and was subsequently discharged home with self-care. The patient had close outpatient follow-up with plastic surgery without any complications.

## Discussion

*Lelliottia amnigena*, previously designated as *Enterobacter amnigenus* in 1981 and renamed in 2013, is a Gram-negative, facultative anaerobic bacillus likely originating from its isolation from aquatic environments [1]. Identified in both water and food sources, this bacterium poses a risk as a potential pathogen, particularly in immunocompromised individuals [8]. The majority of reported *L. amnigena* infections occur in immunocompromised individuals. This includes patients with conditions such as diabetes, cryptogenic cirrhosis, prostatic neoplasms, SARS-CoV-2 infection, chronic obstructive pulmonary disease and bronchiectasis, and those undergoing immunosuppressive therapy [8-11]. As highlighted in case reports, *L. amnigena* has shown pathogenicity by causing bacteremia in heart transplant patients, pneumonia in the ICU, and endophthalmitis [3,9-11]. Moreover, *L. amnigena* has also been implicated in infections among healthy individuals; however, the nidus of infection was trauma-related, as in our patient [11]. Given that infections with* L. amnigena* have occurred in healthy patients, clinical implications should be considered in the differential diagnosis in healthy individuals where there is a history of environmental exposure, such as water and soil, and trauma. It should be noted that *L. amnigena* has also been shown to be a colonizing microbe and not necessarily a pathogen [2,12]. Given this, it is important that clinical context be taken into account.

*Lelliottia amnigena* has been suggested to possess natural resistance to certain classes of antibiotics [13]. Specifically, some reports indicate natural resistance to second- and third-generation cephalosporins, including cefoxitin, cefotaxime, and cefaclor. Conversely, other case reports have shown that *L. amnigena* can be sensitive to antibiotics such as amikacin, cefepime, ceftazidime, ceftriaxone, ciprofloxacin, gentamicin, piperacillin, tobramycin, trimethoprim/sulfamethoxazole, and levofloxacin [13,14]. A key distinction for *L. amnigena* is that some strains, unlike many other *Enterobacter* species, explicitly lack the gene encoding the chromosomal AmpC-type beta-lactamase, rendering them naturally susceptible to all beta-lactam antimicrobials [15]. For our patient, the strain of *L. amnigena* was resistant to ceftriaxone and had intermediate sensitivity to ampicillin; however, it was sensitive to trimethoprim/sulfamethoxazole, piperacillin/tazobactam, ampicillin/sulbactam, and fluoroquinolones.

The case presented is unique due to the patient having no pertinent medical history and subsequently developing an infection with *L. amnigena*, with MRSA and *Corynebacterium*, which caused cellulitis after a traumatic event. The successful surgical and medical management, including debridement, partial amputation, and targeted antimicrobial therapy, highlights the importance of early multidisciplinary intervention and culture-guided treatment in managing complex polymicrobial soft tissue infections. This case expands the known pathogenic potential of *L. amnigena *and emphasizes the need for heightened clinical awareness of atypical pathogens in environmental trauma.

## Conclusions

This case highlights a rare polymicrobial wound infection involving *Lelliottia amnigena*, MRSA, and *Corynebacterium* species following a traumatic lawnmower injury in an otherwise immunocompetent individual. While* L. amnigena *has been previously reported in isolated cases of infection, typically in immunocompromised patients, this case of cellulitis is unique in regard to it being caused by *L. amnigena*,* *which is a rare pathogen, with coinfection of MRSA and *Corynebacterium*, in the context of severe traumatic injury.
